# A Case of Osseous Metaplasia in a Juvenile Rectal Polyp

**DOI:** 10.7759/cureus.59480

**Published:** 2024-05-01

**Authors:** Jonathan H Le, Veronica M Gonzalez

**Affiliations:** 1 Pediatrics, McGovern Medical School at UTHealth Houston, Houston, USA

**Keywords:** intestinal inflammation, mucosal prolapse syndrome, mucosal prolapse, colorectal polyp, osseous metaplasia

## Abstract

Rectal mucosal prolapse is uncommon in children. While most patients present with rectal bleeding and constipation, the occurrence of osseous metaplasia within the prolapsed mucosa is extremely rare. Overlapping clinical, gross, and histological features between rectal mucosal prolapse polyps and malignancy pose a challenge for diagnoses. We describe a case of a 16-year-old male who had a rectal mucosal prolapsed polyp with osseous metaplasia. He initially presented due to periumbilical pain with a sore throat and fever. Incidentally, during the workup of his periumbilical pain, he was found to have a soft tissue mass in his rectum on a CT scan, with a biopsy confirming the diagnosis. The case was complicated by the development of sepsis. The patient was treated with empiric antibiotics and was discharged without further complications.

## Introduction

Rectal mucosal prolapse polyps are rare, benign types of colorectal polyps. The exact etiology of rectal mucosal prolapse polyps is unknown, but it is postulated that they are the result of distortion and twisting of the mucosa caused by repeated colonic spastic contractions leading to mucosal redundancy, venous congestion, and inflammation. This condition is associated with diverticular disease and frequently manifests in patients with a prolonged history of defecation straining. Additional symptoms may include gastrointestinal bleeding, altered bowel habits, hematochezia, and abdominal pain [1].

These polyps pose diagnostic challenges due to their resemblance to other colorectal pathologies, such as inflammatory bowel disease, adenomas, and adenocarcinomas [2]. Here, we describe a case of osseous metaplasia in an unusually large rectal mucosal prolapsed polyp that was further complicated by sepsis.

## Case presentation

**Figure 1:**
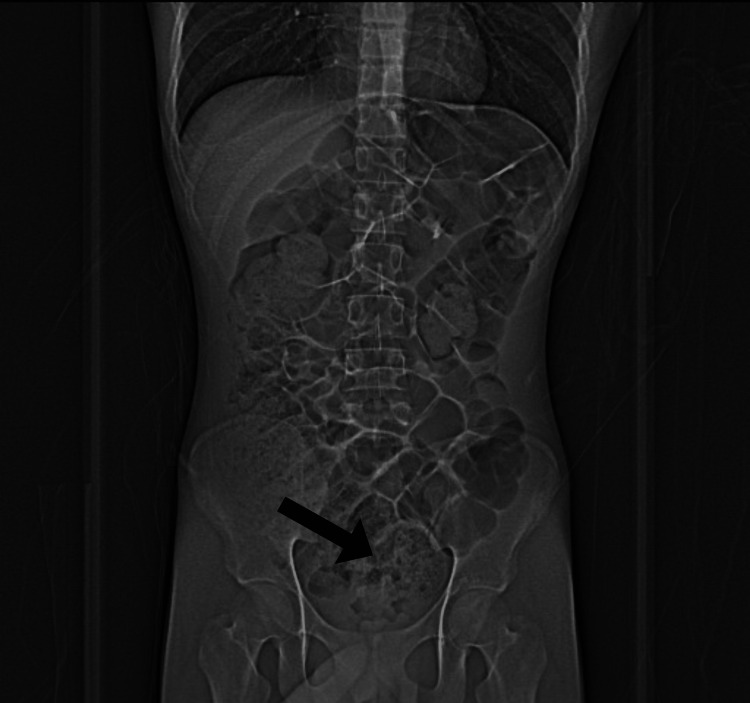
Abdominal computed tomography from outside institution. Heterogeneously enhancing soft tissue (indicated by black arrow) and fluid distending the rectum with very large colonic and rectal stool volume were observed.

A 16-year-old male with no significant past medical history presented as a transfer from an outside institution for a higher level of care with a chief complaint of periumbilical pain with a sore throat and fever. Initial workup at the outside institution showed a CT abdomen/pelvis with heterogeneously enhanced soft tissue and fluid distending the rectum with very large colonic and rectal stool volumes (Figure 1). The patient also reported an unintentional 4.5 kg weight loss over a two-year time period. A review of systems was negative for vomiting, diarrhea, dysuria, constipation, dyschezia, and melena. However, the patient did report small drops of blood in his stool.

On initial physical examination, temperature was 99.6 °F (37.6 °C), heart rate 97 beats/min, blood pressure 105/55 mmHg, respiratory rate 18 breaths/min, and oxygen saturation 97% on room air. His weight was 49.9 kg, with a body mass index (BMI) of 16.8 kg/m2. He was alert, awake, and in no acute distress. The abdomen was soft, non-tender, and non-distended, with no masses or hernias present and normoactive bowel sounds. A rectal exam revealed a 2 cm soft, polypoid, mobile mass felt at the left lateral wall without perianal fissures or fistulas. The remainder of the physical examination was unremarkable.

A complete metabolic panel (CMP), complete blood count (CBC), and inflammatory markers were obtained. Laboratory testing was notable for a hemoglobin 9.9 g/dL, hematocrit 30.4%, mean corpuscular volume (MCV) 73.7 fL, white blood cell (WBC) count 5.9 K/CMM, neutrophils 20%, bands 39.0%, lymphocytes 10%, atypical lymphocytes 8.0%, erythrocyte sedimentation rate 50 mm/hr, C-reactive protein 128 mg/L, procalcitonin 0.17 ng/mL, and a positive fecal occult blood test (Table 1). CMP laboratory values were within normal ranges.

**Table 1:** Laboratory investigations. WBC: white blood cell count; MCV: mean corpuscular volume; ESR: erythrocyte sedimentation rate; CRP: c-reactive protein.

Test	Observed value	Reference range
Hematocrit	30.40%	42.0–54.0%
Hemoglobin	9.9 g/dL	14.0–18.0 g/dL
WBC	5.9 x 10^3^/µL	3.7–10.4 x 10^3^/µL
Neutrophils	20%	45.0–75%
Bands	39%	0.0–11.0 %
Lymphocytes	10%	20–40%
Atypical lymphocytes	8%	≤0.0 %
Platelets	372 x 10^3^/µL	133–450 x 10^3^/µL
MCV	73.7 fL	80.0–94.0 fL
ESR	50 mm/hr	0-15 mm/hr
CRP	128 mg/L	≤2.9 mg/L
Procalcitonin	0.17 ng/mL	0.00–0.10 ng/mL
Occult blood stool	Positive	Negative

**Figure 2:**
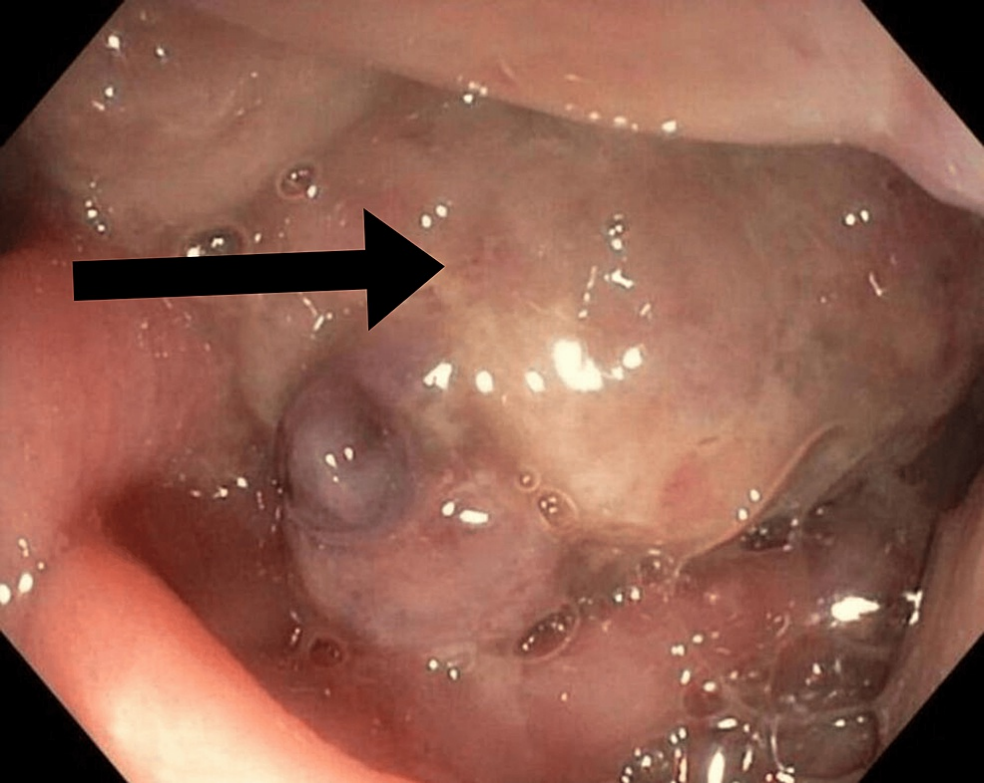
Gross appearance of the rectal mass. The rectal mass (as indicated by the black arrow) measured 5 cm in the longest dimension with multiple pedunculated polyps and surrounding exudates and erythema.

**Figure 3:**
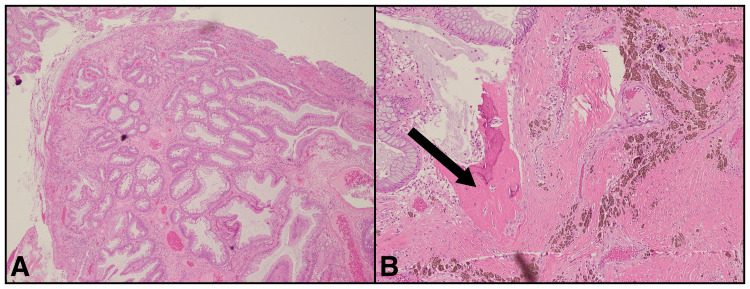
H&E stain of the rectal mass biopsy showing "caps" of ulcerated granulation tissue on the surface with mucin extravasation (A) and foci of osseous metaplasia within the lamina propria as indicated by the black arrow (B).

On admission, a workup was initiated for a rectal mass versus severe proctitis. A colonoscopy performed to evaluate the findings on imaging showed a 5 cm rectal mass with multiple pedunculated polyps with exudates and erythema surrounding the site of the lesion (Figure 2). A biopsy was obtained at this time. The pathology report of the rectal mass revealed hyperplastic rectal mucosa with crypt elongation and dilatation and fibromuscular hypertrophy with an expanded lamina propria. Many “caps” of ulcerated granulation tissue at the surface and mucin extravasation were noted (Figure 3A). Foci of osseous metaplasia were seen within the peri-crypt lamina propria (Figure 3B). The patient subsequently underwent a rectal mass resection due to the mass effect and symptoms.

On postoperative day 2, the patient developed a fever of 102°F (38.9 °C), tachycardia, and hypotension. A CBC and blood culture were obtained to evaluate for possible sepsis secondary to an abscess in the setting of his recent surgical operation. The patient was given two normal saline boluses with improvements in his blood pressure. He was started on piperacillin-tazobactam empirically. Laboratory values were significant for hemoglobin 7.4 g/dL, hematocrit 23.8%, and an elevated WBC count of 21.4 K/CMM with a neutrophilic predominance of 85.9%.

The patient met criteria for severe sepsis with septic shock, requiring transfusion with two units of packed red blood cells and fresh frozen plasma. A rectal exam under anesthesia was performed, and he was found to have a large volume of purulent drainage from a posterior rectal mucosal wound with dehiscence of prior mucosal repairs. The wound was irrigated, a Penrose drain was placed, and metronidazole was added to his postoperative regimen.

The patient subsequently improved clinically with defervescence and a down-trending WBC count. Blood cultures, both preoperatively and postoperatively, remained negative. The patient developed pharyngitis and transient lingual papillitis, which led to poor oral intake post-operatively. By the time of discharge, this was resolved, and he was provided with prescriptions for magic mouthwash and a chloraseptic spray as needed. In addition, the patient was discharged with amoxicillin-clavulanate to complete a total of seven days of therapy from the time the drain was placed. He was instructed to follow up with his primary care provider as well as with pediatric surgery in the clinic for penrose drain removal.

## Discussion

Overall, osseous metaplasia is a rare phenomenon in colonic polyps. To the best of our knowledge, there have been approximately 22 reported cases of osseous metaplasia in juvenile rectal polyps [3-13]. The average patient age at the time of diagnosis was 8.55 years, with the average largest polyp size being 13.68 mm. There have been no reported cases of malignant transformations of these polyps. 

Histologically, rectal mucosal prolapse polyps can be characterized by a spectrum of features that include fibromuscular hyperplasia of the lamina propria, mucosal gland elongation and distortion, serration, inflammatory cell infiltrate, and surface ulceration [14]. Villiform epithelial hyperplasia and regenerative atypia, along with the presence of mucus lakes, may also be observed. These findings can make proper diagnosis challenging due to overlapping symptomatology and histology with other disease processes such as adenomas and adenocarcinomas [2].

The precise cause of osseous metaplasia remains unclear, but a study done by Marks and Atkinson in 1964 suggested that chronic inflammation with disruption of normal crypt architecture may be the cause of osteogenic stimulation of fibroblasts to transform into osteoblasts [15]. More recent studies have suggested that the expression of bone morphogenic proteins (BMPs) in fibroblasts may also play a role in the pathogenesis of osseous metaplasia [16].

Additionally, in our case report, the patient suffered from postoperative septic shock. It is possible that an existing abscess may have predisposed the patient to sepsis during the surgical resection of his polyp. Some evidence of inflammation was present at the time of diagnosis, notably the exudates and erythema surrounding the rectal polyp. It was unclear if there was any significance to the patient's initial presentation of a sore throat and periumbilical pain. Although rectal polyps are known to cause abdominal pain, both it and the sore throat had subsided by the time the patient arrived at our institution. Of note, our patient’s polyp was the largest reported at 50 mm. Its large size may have provided a nidus for infection.

## Conclusions

Rectal mucosal prolapse polyps pose a significant challenge for clinicians to differentiate them from other causes of rectal masses, including malignancy, inflammatory bowel disease, and proctitis, due to their overlapping clinical and histologic features. Although it is relatively rare, osseous metaplasia has no known clinical significance in diagnosing rectal mucosal prolapse polyps. A detailed history and histologic examination should be done to help clinicians rule out potential urgent etiologies of rectal masses and prevent possible complications, such as septic shock, as seen in this case.
